# Beryllium carbonate: a model compound for highest capacity carbon sequestration chemistry†

**DOI:** 10.1039/d4ra07753a

**Published:** 2024-12-23

**Authors:** Gad Licht, Kyle Hofstetter, Stuart Licht

**Affiliations:** a C2CNT LLC A4 188 Triple Diamond Blvd, North Venice FL 34275 USA slicht@gwu.edu; b Carbon Corp 1035 26 St NE Calgary AB T2A 6K8 Canada; c Dept. of Chemistry, George Washington University Washington DC 20052 USA

## Abstract

Beryllium carbonate has the highest capacity to bind and release the greenhouse gas CO_2_ compared to amines, ionic liquids, CaCO_3_ or Li_2_CO_3_. The thermodynamic equilibrium for CO_2_ and BeO from BeCO_3_ is calculated. TGA of BeCO_3_ is used to determine the stepwise mechanism of its CO_2_ release, and the low melting point Li/Sr/BeCO_3_ is demonstrated.

## Abstract 2

Beryllium carbonate is introduced as a model compound, establishing a baseline among the highest CO_2_ capture capacities. The stepwise mechanism of beryllium carbonate's CO_2_ release is determined.

## Graphical abstract

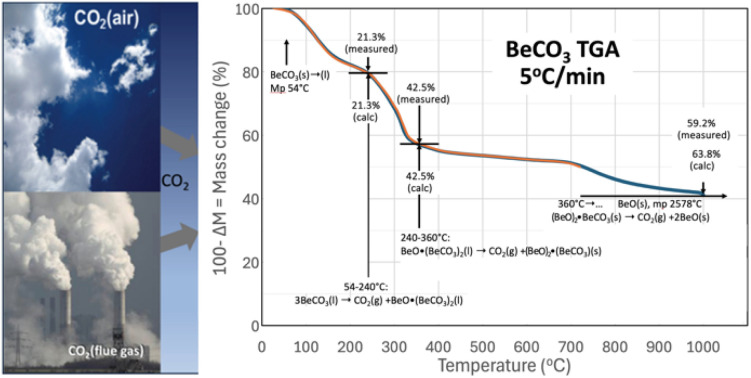

## Introduction

Rising CO_2_ levels are causing catastrophic climate change, and are an existential threat to the planet. Atmospheric CO_2_ levels, stable at 235 (±50) ppm for several hundred thousand years prior to the industrial revolution, have nearly doubled to 424 ppm and continue to rise rapidly. The effects of climate change include an increasing probability of a mass species extinction event.^1^ The ongoing toll on humanity and the planet's habitats are increasingly evident; on a personal note, the first and third authors of this study have just experienced ground zero of two “once in a millennium rainfall events” (Hurricanes Helene and Milton) in a period of two weeks, while the middle author is subject to increasingly frequent forest fires.

A principal path to mitigate climate change is CO_2_ sequestration, and CO_2_ capture capacity is a measure of the extent of a chemical species binding of CO_2_ for use in carbon sequestration chemistry. CO_2_ capture capacity is quantified as mass or mole capacity of CO_2_ per mass or mole of absorbent. In addition to the general need for effective CO_2_ trapping materials to mitigate CO_2_-induced global warming and climate change, other examples of the need for lightest weight carbon capture materials for CO_2_ air scrubbing, include those needed by submersibles, submarines, and spacecraft.^2,3^

Several recent reviews have focused on amines (and amino acids), calcium oxide (to calcium carbonate), and ionic liquids to bind and release CO_2_.^4–9^ More recently, there has been a growing focus on nanomaterials, such as carbon nanomaterials,^10–12^ and also on and lithium carbonate and mixed lithium/strontium carbonates^13–16^ to capture CO_2_ has emerged. Table 1 summarizes common absorbents including amines, ionic liquids, calcium oxide (to calcium carbonate), and more recently, lithium oxide (to lithium carbonate). In this study, beryllium carbonate is introduced as a model compound, establishing a baseline for among the highest capacities of CO_2_ captured.

**Table 1:** The high capacity for CO_2_ of beryllium oxide as beryllium carbonate compared to other absorbents

Chemical absorbent/product	Acronym	Formula	Formula weight, g mol^−1^	CO_2_ storage mechanism	Capacity for CO_2_, mol CO_2_/mol absorbent	Source, reference	Capacity for CO_2_, kg CO_2_ per kg absorbent
1-Butyl-3-methylimidazolium bis(trifluoromethylsulfonyl)imide	[Bmim][Tf_2_N]	C_10_H_15_F_6_N_3_O_4_S_2_	419.37	Ionic liquid	0.68	7	0.07
1-Aminopropyl-3-methylimidazolium tetraborafluorate/various products	APMim[BF_4_]	C_7_H_14_N_3_BF_4_	227.01	Ionic liquid	0.34	7	0.07
1-Butyl-3-methylimidazolium hexafluorophosphate/various products	[Bmim][PF_6_]	C_8_H_15_F_6_N_2_P	284.186	Ionic liquid	0.51	7	0.08
1-Butyl-3-methylimidazolium tetrafluoroborate/various products	[Bmim][BF_4_]	C_8_H_15_BF_4_N_2_	226.03	Ionic liquid	0.44	7	0.09
Taurine to protonated acid, HCO_3_^−^ or carbamate		C_2_H_7_NO_3_S	125.14	Amino acid	0.7	7	0.25
Proline to protonated acid, HCO_3_^−^ or carbamate		C_5_H_9_NO_2_	115.132	Amino acid	0.7	7	0.27
Glycine to protonated acid, HCO_3_^−^ or carbamate		C_2_H_5_NO_2_	75.067	Amino acid	0.6	7	0.35
Monoethanolamine to RNHCOO^−^	MEA	C_5_H_13_NO_2_	61.08	Amine	0.5	7	0.36
Diethanolamine to RNHCOO^−^	DEA	C_4_H_11_NO_2_	105.14	Amine	0.5	7	0.21
Methyldiethanolamine to ‘’”	MDEA	C_5_H_13_NO_2_	119.164	Amine	1	7	0.37
Diethylethanolamine to “”	DEAE	C_6_H_15_NO	117.192	Amine	0.5	7	0.19
Calcium oxide to carbonate		CaO	56.08	CaO + CO_2_ → CaCO_3_	1	9	0.78
Lithium oxide to carbonate		Li_2_O	29.88	Li_2_O + CO_2_ → Li_2_CO_3_	1	16	1.47
Beryllium oxide to carbonate		BeO	24.01	BeO + CO_2_ → BeCO_3_	1	This study	1.83

CO_2_ can be captured and stored by thermal cycling. In this case, dilute CO_2_ is generally introduced at a lower temperature and released in a concentrated form at a higher temperature. Thermal cycling can comprise adsorption chemistry, as generally occurs with various amine carbon capture chemistries,^5,7^ or by chemical reactions, as occurs in the reaction of dissolved or solid calcium oxide with CO_2_ to calcium carbonate, followed by high-temperature decomposition of calcium carbonate back to calcium oxide.^8^ Thermal cycling is often accompanied by pressurization and also by different subsequent processes to sequester (Carbon Capture and Storage, CCS) or chemically convert (Carbon Capture Utilization and Storage, CCUS) the captured concentrated CO_2_. As an alternative to thermal cycling, the CO_2_ capture can be accomplished by electrolysis (such as the electrochemical splitting of CO_2_ to C and O_2_) to a product containing the captured CO_2_, such as the formation of Carbon NanoTubes (CNTs) from CO_2_ in molten carbonates.^17–20^ This latter CCUS process often occurs in a single step.

In Table 1, the capacity for CO_2_ is compiled for common CO_2_ absorbents (absorbents referring to both adsorbents, absorbents, and reactants). The capacity for CO_2_ is presented in units of both mole CO_2_/mole absorbent and also in more typical units of kg CO_2_ captured per kg absorbent in the last column. Pragmatic capacities for CO_2_ will be lower than those compiled when a matrix (such as an inert membrane or solvent stabilizer) is required as an additional mass component in the CO_2_ capture process.

Amines and amino acids have been widely studied both as absorbents, principally in the liquid phase, to absorb and release CO_2_, and as adsorbents principally affixed on membranes to adsorb and release CO_2_. As seen in Table 1, amines and amino acids have respective capacities for CO_2_ of 0.19–0.37 or 0.27–0.35 kg CO_2_ per kg (amine or amino acid). Ionic liquids have attained capacities of 0.07–0.09 kg CO_2_ per kg. CaO/CaCO_3_ has a capacity for CO_2_ of 0.78 kg CO_2_ (determined as of CaO + CO_2_ → CaCO_3_ from the 44.01 g per mol FW of CO_2_ to the 56.08 g per mol FW of CaO). The lighter molecular weight Li_2_O/Li_2_CO_3_ has a capacity for CO_2_ of 1.47 kg CO_2_. In order of increasing CO_2_ capacities, the absorbents are ionic liquids < amino acids & amines < CaO (to CaCO_3_) < Li_2_O (to Li_2_CO_3_).

In this study, beryllium carbonate, with the lowest melting points of inorganic carbonates, is introduced as a model compound establishing a baseline for maintaining the highest capacities for CO_2_ (1.83 kg CO_2_ per kg BeO → BeCO_3_). Beryllium carbonate is also as an example of a melting point decrease facilitator by addition of BeCO_3_ to binary Li/SrCO_3_ electrolyte to become the substantially lower melting point ternary Li/Sr/BeCO_3_. Binary mixtures typically melt at lower temperatures than pure components because the presence of different molecules disrupts the crystal lattice, weakening intermolecular forces and reducing the energy needed to melt, and in this case CO_2_ release can then be achieved using a lower thermal energy input.

## Results and discussion

### Phase changes & CO_2_ equilibria of alkali & alkali earth carbonates

As summarized in Table 2, the melting point of beryllium carbonate (mp 54 °C), Be_2_CO_3_, is low in the extreme compared to that of the other alkali earth or alkali carbonates, or compared to their binary or tertiary carbonate mixtures. It should be noted that reported values of carbonate melting points exhibit a considerable range of 10 °C or more. For example, in 7 studies, the melting point of Li_2_CO_3_ is reported from 700 °C to 728 °C.^22–28^ Furthermore, the CO_2_ concentration in the atmosphere has increased by 35% since the first of these reports appeared (in 1957). The stability of lithium carbonate increases under 1 atm of CO_2_, and we also note that many properties, including melting points, of species related to CO_2_ equilibrium will need to be reevaluated due to the rapidly increasing atmospheric concentration of this greenhouse gas.

**Table 2:** Melting point of alkali and alkali earth carbonates and their eutectic mixtures

Carbonate	Melting point (°C)	Decomposition point (°C)	Ref.
Be_2_CO_3_	54	∼100	21
Li_2_CO_3_, Na_2_CO_3_ or K_2_CO_3_	723, 851 or 891	∼1300^a^ (Li_2_CO_3_)	30
BaCO_3_	810	∼1360	31 and 32
MgCO_3_	—	∼350	32
CaCO_3_	—	∼850	31 and 32
SrCO_3_	1494	1494	13
Li_2_/BaCO_3_; 55/45 mol%	609		31 and 34
K_2_CO_3_/MgCO_3_; 57/43 mol%	460		35
Li_2_/K_2_CO_3_; 62/38 mol%	498		35
Na_2_/K_2_CO_3_; 56/44 mol%	710		35
Li_2_/Na_2_/K_2_CO_3_; 43.5/31.5/25 mol%	397		35
Li_2_/Sr_2_CO_3_; 60/40 wt%	680		13
Be/Sr/Li_2_CO_3_; 33/33/33 wt%	480		This study

a Li_2_CO_3_ decomposition is more rapid under argon than under air.^15^

Alkali carbonate binary mix eutectics have lower melting points. The lowest melting alkali carbonate is generally considered to be the Li_*x*_Na_*y*_K_*z*_CO_3_ with a melting point of 397 °C (at ∼*x* = 0.47, *y* = 0.62, *z* = 0.5), still considerably higher than the melting point of BeCO_3_, by 343 °C.

The extent to which an alkali or alkali earth carbonates retains CO_2_ is given by:

1 MCO_3_ ⇌ CO_2_(gas) + MO (M = Be, Mg, Li_2_, Na_2_, *etc.*)

2 *K* = *p*_CO_2__*a*_MO_/*a*_MCO_3__; *K*(*T*) = e^−Δ*G*(*T*)/RT^

An extensive literature search did not reveal phase diagrams or equilibria for beryllium carbonate. We've calculated the BeCO_3_/BeO + CO_2_ equilibrium from the available enthalpy and entropy of the constituent species.^29^

**Fig. 1:**
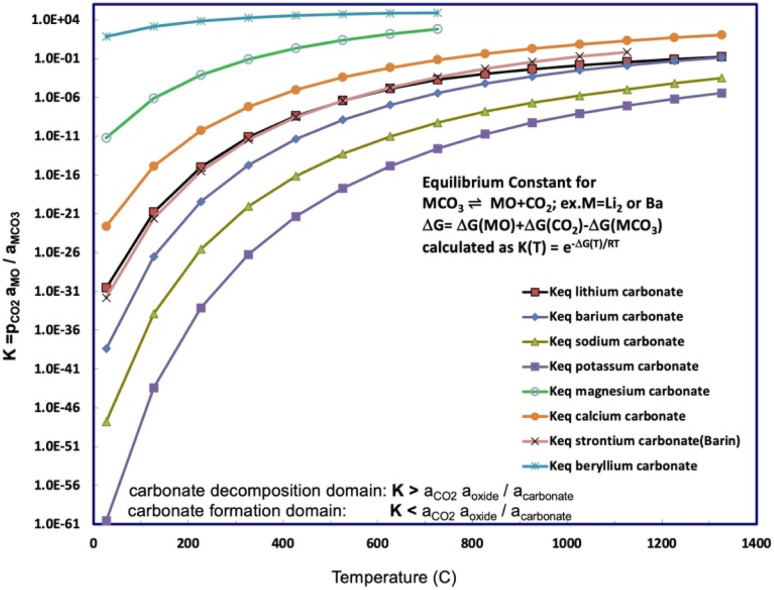
Equilibrium constant for CO_2_ release calculated for beryllium carbonate and compared to a range of alkali and alkali earth carbonates. The equilibrium constants as a function of temperature for strontium, lithium, sodium, potassium, and barium carbonate. The equilibrium constants are calculated from the free energy according to eqn (5). The free energy is calculated from the metal carbonate, metal oxide, and carbon dioxide enthalpies and entropies.^29,36–39^

Fig. 1 presents a comparison of the carbonate/oxide equilibrium constant for binding and releasing of CO_2_ by beryllium carbonate compared to those for alkali, or other alkali earth carbonates as a function of temperature. Below any of the Fig. 1 equilibrium presented curves, that is, in the high CO_2_ activity domain, the carbonate salt will spontaneously form from CO_2_ and the salt's oxide. Above any Fig. 1 equilibrium curve, the low CO_2_ activity domain (*a*_CO_2__*a*_oxide_/*a*_carbonate_ < *K*), the carbonate salt will spontaneously decompose. For example, as noted in Table 2, solid MgCO_3_ decomposes at 350 °C, releasing bound CO_2_, and as seen is the second largest (other than BeCO_3_) of the eqn (2) carbonate/oxide equilibrium constants. The high industrial carbon footprint conversion process of limestone to lime or cement depends on the solid state decomposition of calcium carbonate, such as aragonite, which occurs at ∼850°.

Li_2_CO_3_/Li_2_O was introduced as among the highest CO_2_-capture materials, as delineated in the next to last row of Table 1, with a storage capacity of 1.47 kg CO_2_ per kg Li_2_O. Under argon, Li_2_CO_3_ entirely dissociates to CO_2_ + Li_2_O. Specifically, at a TGA rate of 5° min^−1^ under 1 atm of argon Li_2_CO_3_ dissociation starts around the lithium carbonate melting point of 723 °C, and is 98% complete to Li_2_O by 900 °C, and under 1 atm of pure CO_2_ also starts at 723 °C, but the dissociation is less than 10% complete by 900 °C.^15^ However, under even small partial pressures of CO_2_, such as the 426 ppm (and rising) of atmospheric CO_2_, Li_2_CO_3_ only fractionally dissociates to CO_2_, attaining 0.3 molal concentration Li_2_O per kg molten Li_2_CO_3_ at 750 °C.^36^ This high capacity was experimentally realized in the form of the electrolytic splitting of CO_2_ in molten Li_2_CO_3_ to graphene nanocarbons.^40–49^ The small concentration of dissolved Li_2_O in molten Li_2_CO_3_ under air is sufficient to support high CO_2_ splitting electrochemical current and continuous renewal of the molten Li_2_CO_3_ electrolyte with CO_2_.^50^

The relationship between melting and decomposition temperatures for carbonates is complex. Beryllium carbonate has respective melting and decomposition points of BeCO_3_ (*M*_p_ = 54 °C and *D*_p_ = 100 °C),^21^ lithium carbonate Li_2_CO_3_ (*M*_p_ = 723 °C and *D*_p_ = 1300 °C), while as seen in Table 2, solid calcium and barium carbonate do not melt nor sublime, but rather decompose directly to calcium or barium oxide and carbon dioxide, finally, strontium carbonate has equivalent, but very high melting and decomposition points SrCO_3_ (*M*_p_ = *D*_p_ = 1494 °C). For binary and ternary mixtures, all the higher melting point carbonates are observed to be highly soluble in lithium carbonate. For example, over 60 wt% SrCO_3_ is miscible in 750 °C molten Li_2_CO_3_.^13^

The temperature at which individual carbonates do, or do not, melt is observable and reproducible to within a few degrees. However, the decomposition point is much less distinct, occurring over hundreds of degrees. For example, while the formal Li_2_CO_3_ decomposition point in Table 2 is ∼1300, substantial decomposition has already occurred at 750 °C with release of CO_2_ and the resultant Li_2_O forming as a dissolved salt within the molten Li_2_CO_3_.^36^ Hence, this study focuses on the more precise melting point, rather than the broad range of observable decomposition point temperatures.

### Beryllium carbonate as an ultra-high CO_2_ storage material

When modeled as a specific example of eqn (1), beryllium carbonate is a high-capacity carbon capture storage material. Carbon dioxide is stored in beryllium carbonate and is released in the reaction to CO_2_ and beryllium oxide:

3 BeCO_3_ ⇌ CO_2_(gas) + BeO

In accord with eqn (3), BeCO_3_ (FW 69.02 g mol^−1^) stores 44.01 g mol^−1^ CO_2_/BeO (FW 25.01 g mol^−1^) = 1.83 kg CO_2_ per kg BeO → BeCO_3_, as included in Table 1. As seen in Fig. 1, beryllium carbonate provides the largest of the equilibrium constants to release CO_2_ of any of the alkali earth or alkali carbonates, and will begin to release CO_2_ near ambient temperatures. At room temperature, beryllium carbonate (solid BeCO_3_) is often stored under a blanket of CO_2_ gas to prevent its decomposition. Beryllium oxide is the lightest weight oxide (other than water), and lithium and beryllium oxide have among the highest carbon capture storage capacities (as moles CO_2_ storable per kg oxide).

Thermodynamically, BeCO_3_ is the carbonate best suited to initiate storage and release of CO_2_ at low temperatures. BeCO_3_ is less prevalent as a salt than calcium, lithium, or strontium carbonate. Be is only the 48th most abundant element in the earth's upper crust,^51^ and it and its oxide, particularly in powder form, is carcinogenic. However, the storage of CO_2_ by BeCO_3_ serves as a model for among the highest carbon storage capacity materials and lowest mass CO_2_ scrubbers.

### The stepwise mechanism of beryllium carbonate CO_2_ storage

Eqn (1) only provides a thermodynamic overview of a carbonate's capability to release CO_2_. The individual steps in the process of the binding of CO_2_ into beryllium carbonate are investigated here by ThermoGravimetric Analysis, TGA.

**Fig. 2:**
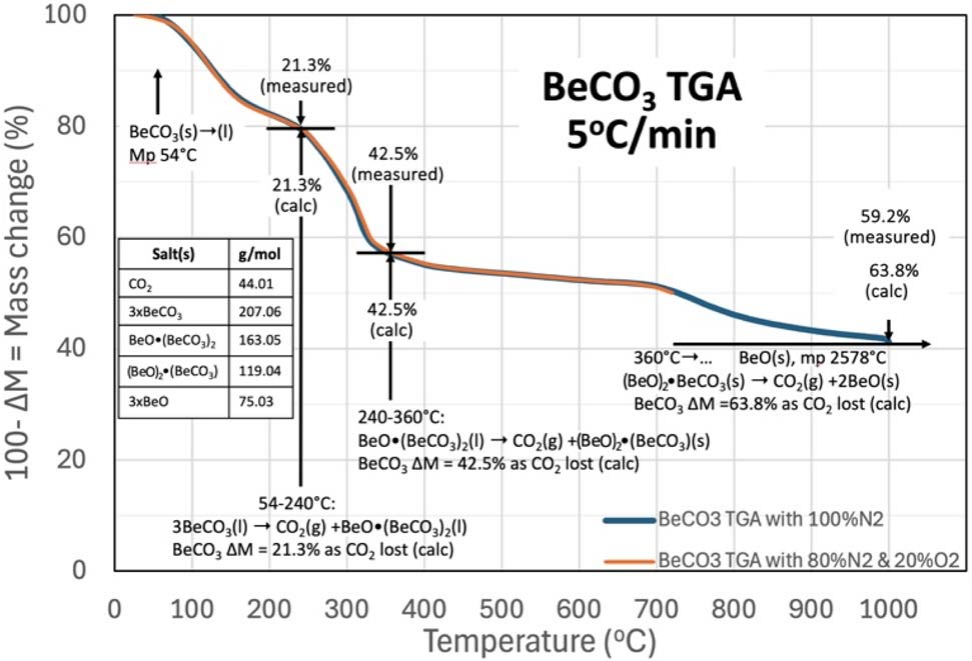
TGA analysis of beryllium carbonate. The TGA is conducted from 30 °C with a 5 °C increase per minute in either (1) 80% N_2_/20% O_2_ (from 30 °C to 730 °C) or (2) 100% N_2_ (from 30 °C to 1000 °C). Note, the observed TGA are identical (from 30 °C to 730 °C) in either the O_2_ or pure N_2_ environments.

Fig. 2 presents the TGA results of beryllium carbonate conducted from 30 °C, with a 5 °C temperature increase per minute, and in atmospheres of either (1) 80% N_2_/20% O_2_ gas mix shown in the orange curve from 30 to 730 °C or (2) 100% N_2_ shown in the blue curve from 30 °C to 1000 °C. In the figure, the downward trend in the mass is seen to start at approximately, the cited^21^ 54 °C melting of BeCO_3_. The equivalence of the curves with or without an atmosphere containing O_2_ provides primary evidence that O_2_ is neither evolved nor absorbed by beryllium carbonate during the TGA, and that species in equilibrium with O_2_, including oxides, peroxides, and superoxides, those species are not participants in reactions related to the TGA temperature sweep.

In Fig. 2 above 54 °C, BeCO_3_ rapidly evolves CO_2_ upon melting (at increasing temperature, the release of CO_2_ from BeCO_3_ is an exergonic, spontaneous reaction). Released gases diffuse more slowly through a solid than through a liquid.^52^ Salts evolving CO_2_ from the liquid, as opposed to from the solid form, facilitate the rapid release of CO_2_. For example, comparing liquid and solid CO_2_ amine sorbents, solid gas reactions require much higher minimum work,^53^ and the concurrent observed increased rate of mass loss acts as an indicator that the salt has melted. In solid salts that can release CO_2_ by decomposition, CO_2_ release is constrained by surface depletion and by the slow diffusion of CO_2_ to the solid surface. Whereas, in the molten state, the liquid surface is continuously replenished, sustaining facile CO_2_ access to the surface, and to the interior liquid bulk. In the figure, the mass loss and temperature are noted at the start of rapid mass declines with increasing temperature, and a mechanism of CO_2_ mass loss is then determined by calculating mass consistent changes of the equivalent calcinated beryllium oxide and BeCO_3_ salts.

As seen in Fig. 2, molten BeCO_3_ evolves CO_2_ to become BeO·(BeCO_3_)_2_ from ∼54 °C to 240 °C, consistent with the equivalence of both the observed and the calculated mass loss as mass loss (of CO_2_/mass BeCO_3_) of 21.3% when one CO_2_ is evolved from 3 BeCO_3_ to become BeO·(BeCO_3_)_2_, and the rapid mass loss indicative of facile CO_2_ evolution from a liquid. In the future, several orders of magnitude larger than the TGA mg size samples would be useful to visually corroborate that this is in the liquid (l) phase at these temperatures:

4 3BeCO_3_(l) → CO_2_(g) +BeO·(BeCO_3_)_2_(l) *T* = 54–240 °C

At increasing temperature, the molten BeO·(BeCO_3_)_2_ then evolves CO_2_ to become (BeO)_2_·BeCO_3_ (FW 119.04) from 240 °C to 360 °C; again, as determined by the equivalence of both the observed and the calculated mass loss of 42.5% when 2CO_2_ are evolved from 3BeCO_3_ to become BeO·(BeCO_3_), and once again the rapid mass loss indicative of facile CO_2_ evolution from a liquid:

5 BeO·(BeCO_3_)_2_(l) → CO_2_(g) +(BeO)_2_·(BeCO_3_) (s) *T* = 240–360 °C

From 360 °C to ∼700 °C, there is an observed slow, steady rate of CO_2_ evolution as the mass loss observed in Fig. 2 increases to 50.9% from the original BeCO_3_. The slow rate of CO_2_ evolution is evidence that the eqn (5) product may be solid, and the additional (50.9–42.5%) 8.4% mass loss from 3BeCO_3_ is evidence that the 2BeO·(BeCO_3_) has evolved an additional 0.4 CO_2_ over this temperature range with either a lower thermodynamic drive to release CO_2_, or has reverted to the solid phase.

6a (BeO)_2_·(BeCO_3_)(s) → 0.4CO_2_(g) +(BeO)_2.4_·(BeCO_3_)_0.6_(l) *T* = 360–700 °C

Equivalent to integral molecular values of:

6b 5(BeO)_2_·(BeCO_3_)(s) → 2CO_2_(g) +(BeO)_12_(BeCO_3_)_3_(l) *T* = 360–700 °C

Above 700 °C, the observed rate of mass loss and CO_2_ evolution again increased, indicative that the product has once again entered a liquid phase as noted on the right side of eqn (6a). In eqn (6b) (BeO)_12_(BeCO_3_)_3_(l) is a generalization of the total equivalence of BeO and BeCO_3_ in the product, and it is likely that this consists of a solid BeO (mp 2578 °C) in a liquid phase of mixed BeO_*x*_/BeCO_3_. This product then evolves CO_2_ to become BeO(*s*) from ∼700 °C onward. As the end product of the beryllium carbonate CO_2_ loss is a solid, high melting point BeO (mp 2578 °C), and by 1000 °C, the mass observed mass loss has reached 59.2% of the full, calculated 63.8% CO_2_ mass loss from BeCO_3_. Holding the TGA temperature at 1000 °C for 4 more hours resulted in a further mass loss of 1.2% to 60.4% of the full, calculated 63.8% CO_2_ mass loss from BeCO_3_:

7 (BeO)_2.4_·(BeCO_3_)_0.6_(l) → 3BeO(*s*); *T* = 700 °C to *T* > 1000 °C

For an overall reaction of:

8 3BeCO_3_(l) → 3CO_2_(g) +3BeO; *T* = 54 °C to *T* > 1000 °C

The thermal release of CO_2_ from BeCO_3_ does not result in the formation of powdered BeO, which can be toxic, but rather initially forms BeO_*y*_·(BeCO_3_)_*y*_, and then at highest levels of CO_2_ release temperatures, forms BeO in the TGA as a sintered (solid) mass due to the high temperature of formation, rather than an easily dispersible and potentially toxic powder.

The kinetically and thermodynamic-driven release of CO_2_ by heating BeCO_3_ and beryllium oxide/carbonates intermediate compounds has been demonstrated, and will presumably similarly occur by alternatively reducing the pressure over those compounds, or by simultaneously heating and reducing the pressure of those compounds. Thermodynamically, the storage of CO_2_ by beryllium oxide and beryllium oxide/carbonate intermediates is energetically favored by the reverse process of cooling or pressurizing under CO_2_ beryllium oxide and beryllium oxide/carbonate intermediates and stores CO_2_. Beryllium oxide is stable, and this stability to reaction can be overcome by introducing kinetic facilitation to increase the rate of CO_2_ uptake by cooling and/or with pressurized CO_2_.

Future studies can probe the likelihood that the reverse beryllium oxide reaction with CO_2_ to beryllium carbonate can be facilitated by means including: (i) bubbling CO_2_ through the various molten (liquid phase) stages of beryllium oxide and its beryllium oxide/carbonate intermediates or forming a liquid aerosol combined with CO_2_, (ii) increasing the surface area of the various solid phases stages of beryllium oxide and its beryllium oxide/carbonate intermediates such as by forming a powder, solid aerosol or fixing it to a high surface membrane or aerogel while combining with CO_2_, (iii) introducing the CO_2_ by mixing with a combined solid and liquid phase (slush) of beryllium oxide/carbonate intermediates, (iv) or a multistep reaction to incorporate CO_2_ into beryllium oxide such as, but not limited to, the (iva) the facile reaction of CO_2_ with ammonium compounds to form ammonium carbonates and the (ivb) reaction of beryllium oxide with sulfate compounds to form beryllium sulfates, followed by the (ivc) the facile reaction of ammonium carbonates and beryllium sulfates to form BeCO_3_.

### Beryllium-induced carbonate electrolyte melting point decrease

Li_2_CO_3_ is expensive due its relative scarcity and due to its increasing demand as a primary resource for EVs, but is useful for CO_2_ removal and its electrolytic transformation to graphene nanocarbons.^16^ There is less demand for SrCO_3_ and its derivative salts, such as SrO, are also much more abundant, and an order of magnitude less expensive than Li_2_CO_3_.^13,51^ We had demonstrated that molten Li_2_CO_3_ based electrolytes are effective for CO_2_ carbon capture by the electrolytic splitting of CO_2_. Interestingly, we recently found that the replacement of the majority of the Li_2_CO_3_ by SrCO_3_ and SrO is also effective. The low-Li_2_CO_3_ electrolytes based on SrCO_3_ are substantially less expensive than comparable Li_2_CO_3_-based electrolytes, and are useful for splitting and transforming CO_2_ to stable graphene nanocarbons including CNTs and carbon nano-onions (ESI†).^13^ The use of an electrolyte that is a binary mixture (for example, Sr/Li carbonate or SrO/Li_2_CO_3_) that can provide a low melting point electrolyte that facilitates transition metal nucleated growth from CO_2_ of nanographene carbon allotropes is preferred.^13,16,40,42,47^ Low-Li_2_CO_3_ electrolysis may be performed using a planar, rather than a coiled, and brass, rather than Monel, cathode without substantially affecting low-lithium CNT growth from CO_2_.^13,40,41^

**Fig. 3:**
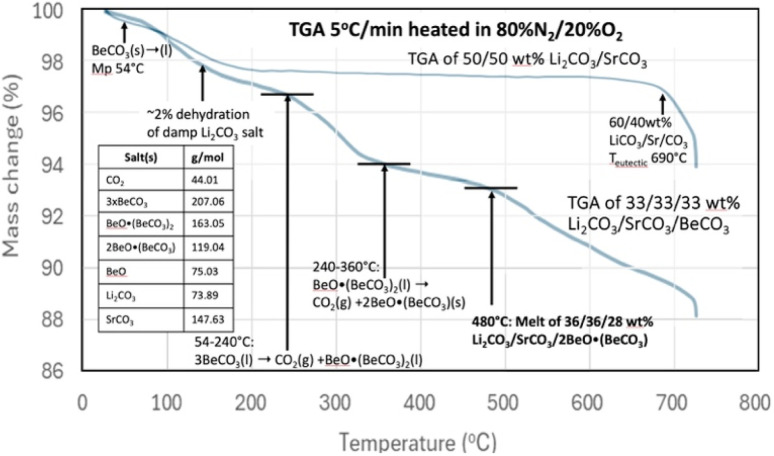
TGA analysis of 50/50 wt% Li_2_CO_3_/SrCO_3_ compared to 33.3/33.3/33.3 wt% Li_2_CO_3_/SrCO_3_/BeCO_3_. The TGA is conducted from 30 °C to 730 with a 5 °C increase per minute in 80% N_2_/20% O_2_.

Pure SrCO_3_ has a high melting point of 1194 °C, and in accord with Table 2 does not decompose until temperatures ≫1000 °C. As previously noted and as measured by TGA, the rapid decomposition of pure Li_2_CO_3_ commences near the 723 °C melting under conditions of no CO_2_ (argon) up through pure CO_2_.^15^ A binary SrCO_3_/Li_2_CO_3_ mix has a melting point of 690–790 °C. The melting point increases as the weight percent of SrCO_3_ in the binary mix increases from 40 to 65%. The eutectic containing 40 wt% SrCO_3_ melts at 690 °C, while the binary 50% SrCO_3_ mix melts at 695 °C (ESI†). Fig. 3 demonstrates that this binary mix melting point is substantially decreased by the inclusion of BeCO_3_ in a ternary mix. Specifically, Fig. 3 compares the TGA's of a binary mix 50/50 wt% Li_2_CO_3_/SrCO_3_ to that of a ternary mix composed one-third by weight each in Li_2_CO_3_, SrCO_3_, BeCO_3_. The TGA starts from 30 °C, with a 5 °C min^−1^ temperature increase, and under an 80% N_2_/20% O_2_ gas mix.

For the TGA of the binary mix of 50/50 wt% Li_2_CO_3_/SrCO_3_, a few percent weight loss is evident in Fig. 3 at low temperature as the damp material dries. Then the mass is moderately constant, decreasing slowly until a more rapid weight loss occurs around the 690 °C melting point observed for a Li_2_CO_3_/SrCO_3_ mixture. Alternatively, in addition to the low-temperature drying, the 33.3/33.3/33.3 wt% Li_2_CO_3_/SrCO_3_/BeCO_3_ ternary mix exhibits the hallmarks of pure BeCO_3_ up to a temperature of 360 °C that were seen in Fig. 2. However, in addition, another sharper decrease in mass loss is observed starting at 480 °C. These are attributed to the melting point of new lower melting ternary mixes of Li_2_CO_3_/SrCO_3_/BeO(BeCO_3_)_2_ and specifically of 36/36/28 wt% Li_2_CO_3_/SrCO_3_/BeO when taking into account the loss of CO_2_ up to 480 °C from the original BeCO_3_ in the formation of BeO. Note, these wt% masses refer to the measured ratio of masses, as distributed through homogeneous speciation in the oxide dissolved in alkali earth carbonate melt, and not that there is a specific release of CO_2_ from an isolated alkali earth carbonate within the liquid.

The melting point observed for the Li_2_CO_3_/SrCO_3_/BeCO_3_ ternary carbonate mix at 480 °C in Fig. 3 is 215 °C lower than the binary Li_2_CO_3_/SrCO_3_mix without the beryllium carbonate addition. Hence, inclusion of BeCO_3_ can lower the melting point of conventional inorganic carbonates prepared without a mix of BeCO_3_.

## Experimental

### Thermodynamic carbonate/CO_2_ equilibrium calculation

Enthalpies, entropies of species *i*, *i* = alkali and alkali earth carbonates, oxides, and CO_2_ are from standard Barin, NIST (calculated from the available condensed phase thermochemistry data Shomate equations), and NASA data bases^.^^29–39^ At any given temperature, the free energy of species “*i*” was calculated as:

9 Δ*G*_*i*_(*T*) = Δ*H*_*i*_(T) − *T*Δ*S*_*i*_(*T*)

The free energy of equilibrium eqn (1) was then calculated as:

10 Δ*G***_eqn (1)_**(*T*) = Δ*G*_CO_2__(*T*) + Δ*G*_MCO_(*T*) − Δ*G*_MCO_3__(*T*)

The eqn (1) equilibria constants for the various alkali and alkali earth carbonates were then calculated in accord with eqn (2).

### TGA measurement, CO_2_ evolution, and phase change

BeCO_3_, 99+% purity, was from Chemsavers. Li_2_CO_3_ was purchased at a battery grade >99.5%, and used as received. The Li_2_CO_3_ had an analyzed composition of 99.8% (Li_2_CO_3_, Shanghai Seasongreen Chemical Co). The SrCO_3_ used was 99.4% pure SrCO_3_ (Shendong Zhi Chemical Co. Thermal) gravimetric analysis (TGA) was conducted using a PerkinElmer STA 6000 TGA/DSC TGA, under either pure N_2_ or a mix of 80% N_2_ and 20% O_2_. TGAs were conducted with 15 mg of sample using a temperature ramp of 5 °C min^−1^ over the indicated temperature range. The daily reproducibility of known pure carbonate or graphene samples served as instrumental calibration, and any buildup of residual carbonate was removed by acid wash. In this study, the similarity of the measured mass change with or without the presence of O_2_ was considered indicative that O_2_ and related species were not participants in the mass loss sequence. TGA rapid mass loss was considered as gas evolved from the liquid phase, while a low rate of mass loss was considered an indicator of gas evolved from the solid phase as delineated in the text.

## Conclusions

Rising levels of CO_2_ in the atmosphere are driving catastrophic climate change, posing an existential threat to the planet. As CO_2_ concentrations increase, they contribute to global warming, extreme weather events, and ecological disruptions. Addressing this challenge requires innovative solutions, one of which is CO_2_ capture and sequestration.

The capacity for CO_2_ is a crucial metric in assessing the effectiveness of various chemical species in absorbing, adsorbing or reacting CO_2_ for sequestration purposes. This capacity is quantified as the amount of CO_2_ (in kilograms) that can be captured per kilogram of the absorbent material. Common absorbents used in this field include amines, ionic liquids, and calcium oxide, which can transform into calcium carbonate. More recently, lithium oxide (Li_2_O), which converts to lithium carbonate (Li_2_CO_3_), has gained attention as a potential absorbent.

In this study BeCO_3_ has been introduced as a model compound due to its remarkably high CO_2_ capture capacity. Although the practical application of beryllium is limited by its scarcity-ranking, as only the 48th most abundant element in the Earth's upper crust, it boasts a CO_2_ capture capacity of 1.83 kg CO_2_ per kg of BeO. This is significantly higher than that of other common absorbents: amines range from 0.19–0.37 kg CO_2_ per kg, ionic liquids capture between 0.07 and 0.09 kg CO_2_ per kg, calcium carbonate (CaCO_3_) with a capacity of 0.78 kg CO_2_ per kg, and lithium carbonate (Li_2_CO_3_) that captures 1.47 kg CO_2_ per kg Li_2_O.

To better understand the thermodynamics involved, the equilibrium between CO_2_ and beryllium oxide (BeO) derived from BeCO_3_ has been calculated and compared to various alkali and alkaline earth carbonates. Thermogravimetric analysis (TGA) of BeCO_3_ has also been conducted to elucidate the stepwise mechanism of CO_2_ release, providing insights into how this process in a stepwise release of CO_2_ at increasing different temperatures.

Additionally, the influence of BeCO_3_ on the melting point of mixtures has been explored. A comparison of the binary carbonate system consisting of Li_2_CO_3_ and strontium carbonate (SrCO_3_) with a ternary system that includes BeCO_3_ illustrates how the addition of BeCO_3_ can substantially depress the melting point. BeCO_3_ has been presented as a model carbonate to advance the foundation of understanding of the requirements of maximum carbon sequestration. It should be emphasized that beryllium, beryllium carbonate and beryllium oxide are more toxic, less abundant and therefore less available and more expensive than previously studied lower sequestration capacity lithium, magnesium and calcium compounds. This research not only highlights the unique properties of BeCO_3_, but also contributes to the broader understanding of CO_2_ capture technologies and their potential role in mitigating climate change.

## Data availability

The data supporting this article have been included as part of the ESI.†

## Author contributions

G. L. and S. L. designed the research; K.·H., G. L. and S. L. performed the research and analysed the data; G. L. and S. L. wrote the paper.

## Conflicts of interest

There are no conflicts to declare.

## Supplementary Material

RA-014-D4RA07753A-s001
