# Supplementary material for: Beryllium carbonate: a model compound for highest capacity carbon sequestration chemistry
Source: RSC Adv. 2024 Dec 23;14(54):40133–40. doi: 10.1039/d4ra07753a (PMC11664358; doi:10.1039/d4ra07753a)
Supplement: RA-014-D4RA07753A-s001 [file RA-014-D4RA07753A-s001.pdf]

Supplementary Information (SI) for RSC Advances.
This journal is © The Royal Society of Chemistry 2024

Supplementary Information accompanying:

Beryllium carbonate: a model compound for highest capacity carbon sequestration chemistry

Gad Licht, Kyle Hofstetter and Stuart Licht
George Washington University, Washington, DC, USA,
Carbon Corp, Calgary, Canada & C@CNT LLC, Florida, USA

In 2015, it was shown that the growth of transition metal nuclei during carbon dioxide electrolysis in lithium carbonate, Li_2CO_3 (mp 723°C) leads directly to the conversion of CO_2 into pure graphene nanocarbons, including carbon nanofibers and carbon nanotubes (CNTs) and later by control of the electrolysis conditions including the composition of the electrodes, temperature current density and the addition of oxides to the electrolytes into carbon nano-onions (CNOs), and a variety of graphene nanocarbon allotropes [1-4]. This transformation of the greenhouse gas CO_2 into valuable GNC products offers a chance to convert CO_2 into a form of carbon stabilized by graphene, thus aiding in mitigating climate change. Graphite is an analogous macroscopic form of layered graphene, and as a mineral graphite has an established geologic (hundreds of millions of years) lifetime.

In 2024 it was shown that $SrCO_3$, while maintaining solidity to 1494°C, is highly soluble in lower temperature molten Li_2CO_3 (Fig. S1), and that its binding of CO_2 (is strikingly similar to that of Li_2CO_3 [5], and substantially unlike that of the other alkali and alkali earth metal carbonates (Fig. 1 main text). This is of consequence as it provides a "Goldilocks" zone of CO_2 to carbonate formation dissociation facilitating the direct electrolytic transformation of graphene nanocarbon allotropes. This includes carbon nanotubes (Fig. S2 to S4) and carbon nano-onions (Fig. S5) grown in $SrCO_3$ binary and ternary electrolytes. The cost of Li_2CO_3 is high due to its concurrent demand for Li-ion battery applications including EVs, while $SrCO_3$ cost is an order of magnitude lower providing for low cost molten carbonate CCUS of CO_2 to its graphene nanocarbon products.

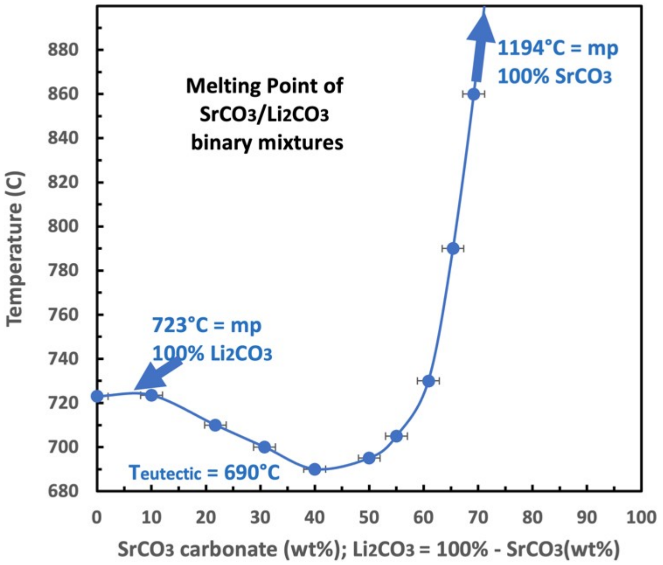

Figure S1. The melting point and high $SrCO_3$ solubility of binary mixtures of $SrCO_3$ and Li_2CO_3. CC licensee open source reference [5].

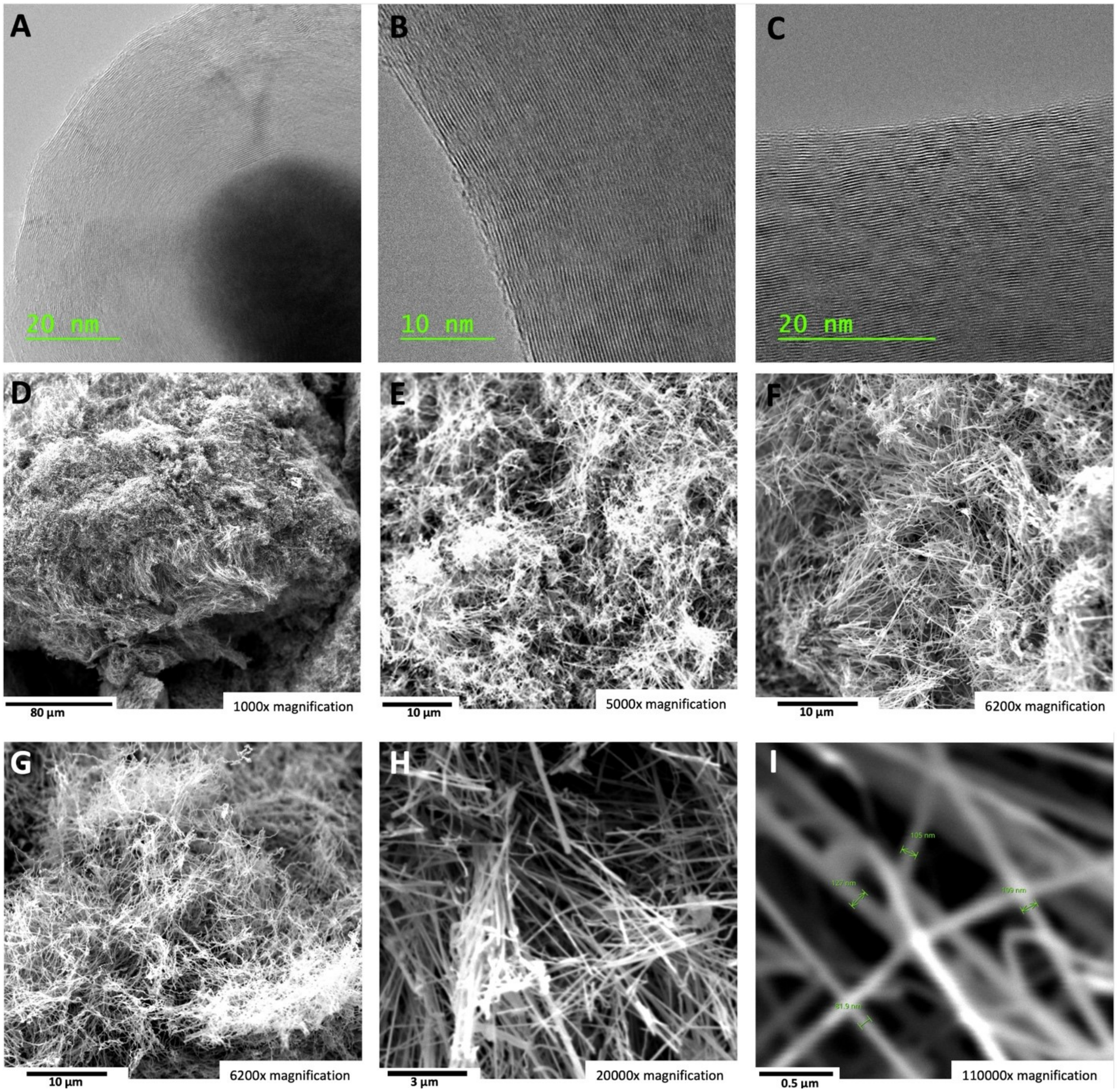

Figure S2. The carbon nanotube product of high current electrolysis in a 45 wt% $SrCO_3$ in a Li_2CO_3 electrolyte. SEM images of the product from 4-hour electrolysis conducted at 790°C and J = 0.6 A/cm^2 with a stainless steel 304 anode at a 120 cm^2 area brass cathode. (A-C) TEM with 20 nm (A&C) and 10 nm (B) scale (3.5 to 7 million x magnification) showing (A) the distinctive hemispherical concentric graphene layers surrounding the transition metal nucleation catalyst and (B and C) and the concentric graphene adjacent layers above and below the hollow CNT core below the CNT tip. SEM magnification is D: 1,000x, E: 5,000x, F, G: 6,200x, H: 20,000x, or I: 110,000x magnification. CC licensee open source reference [5].

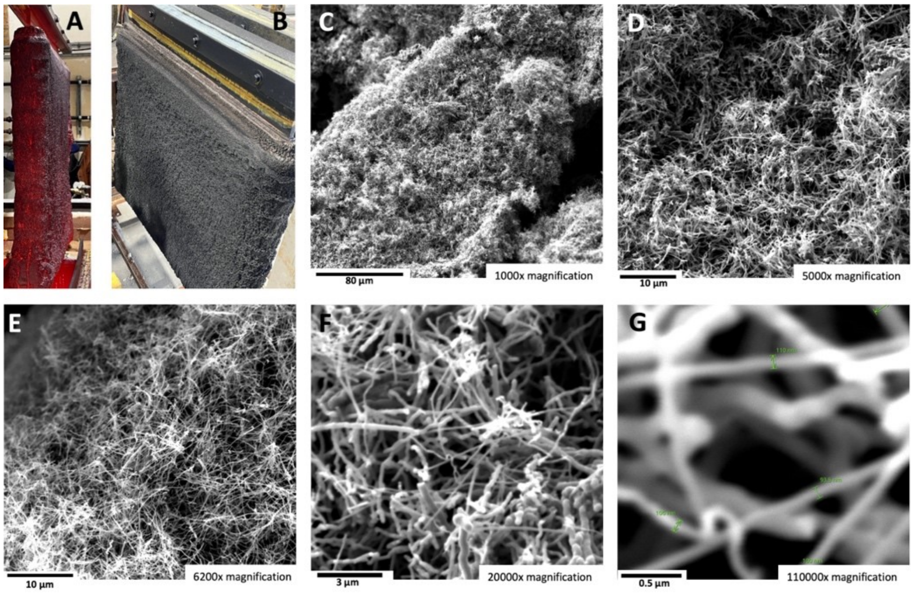

Figure S3. Carbon nanotube product with a high current density, large-area cathode, and 50 wt% $SrCO_3$/50 wt% Li_2CO_3 electrolyte. Panels A and B show the cathode, with an active area of 11,000 cm^2, upon lifting from the electrolyte and subsequent cooling. A: Electrolysis is conducted at 770°C and J = 0.6 A/cm^2 utilizing the stainless steel 304 carbon pot as the anode. The SEM product magnifications are as follows: C: 1,000x, D: 5200x, F: 5,400x, G: 5,500x, H: 10,000x, I: 19,500x, J & K: 45,000x magnification. CC licensee open source reference [5].

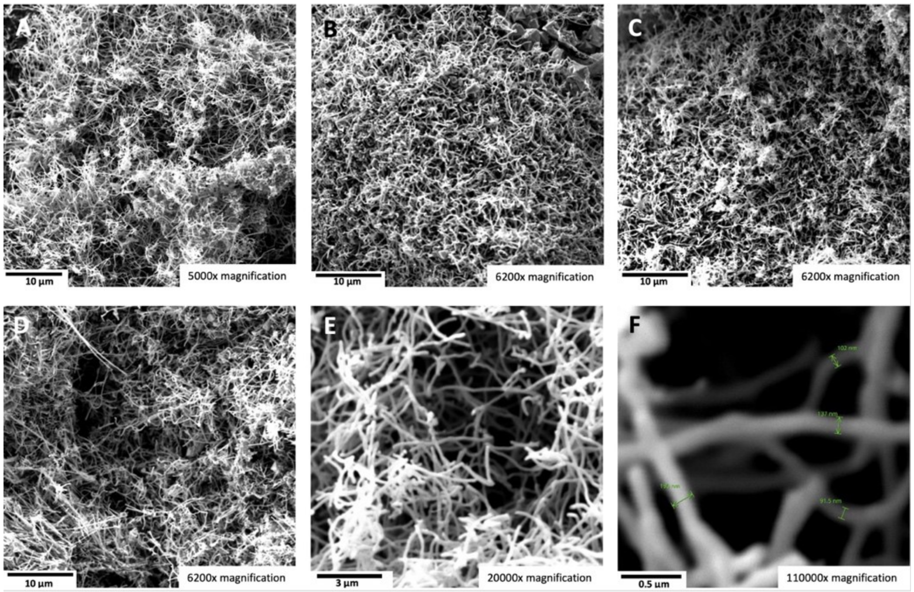

Figure S4. The carbon nanotube product of an electrolysis in a ternary 30 wt% Li_2CO_3 electrolyte with 60wt% $SrCO_3$ and 10 wt% SrB_4O_7 electrolyte. A-E: SEM of the product from the 4-hour electrolysis conducted at 770°C and J = 0.6 A/cm^2 with a stainless steel 304 anode at a 96 cm^2 area brass cathode.

The SEM magnification is: A: 5,000x, B-D: 6,200x, E: 20,000x, or F: 110,000x magnification. CC licensee open source reference [5].

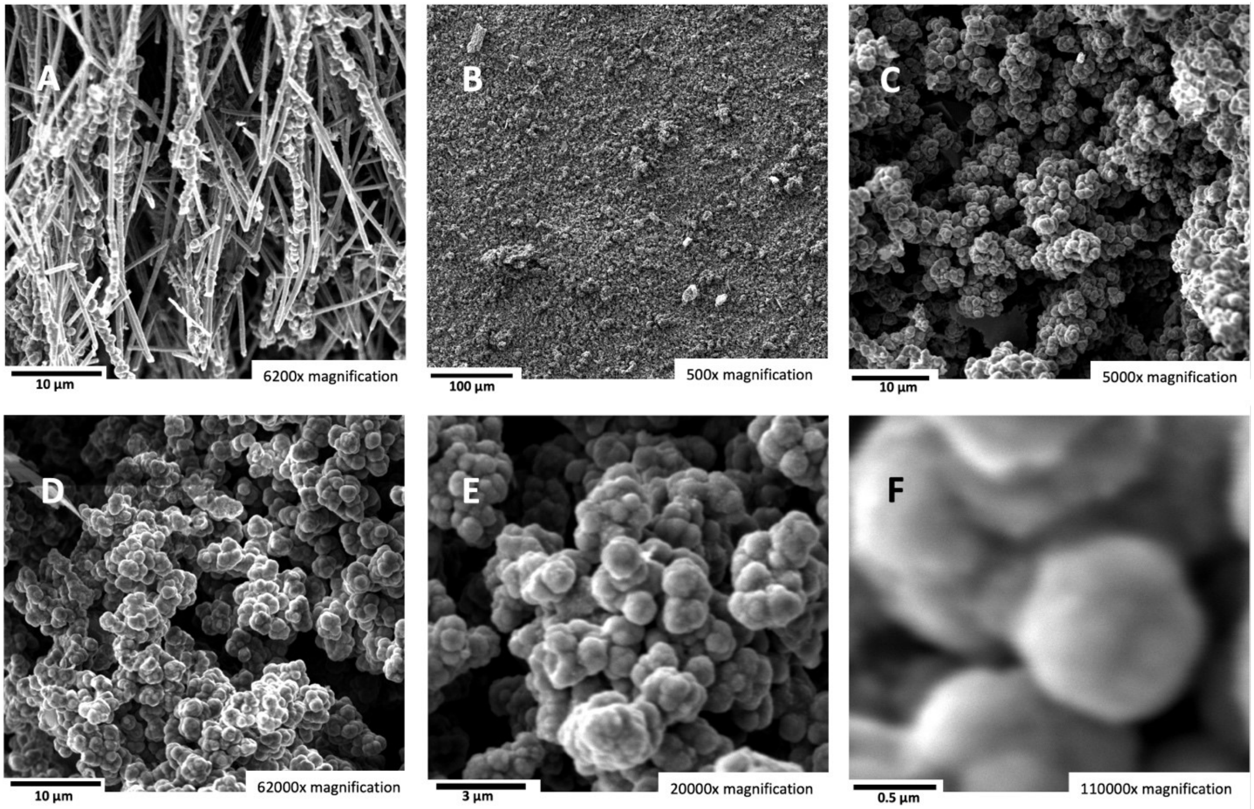

Figure S5. Strontium with lithium and sodium carbonate electrolyte produces carbon nano-onions. Panel A shows an SEM image of a lower CNO product purity previously grown in the same electrolyte, which shows a mixture of CNTs, CNO, and carbon nano-bamboos. Panels B through G show the SEM images of a product with a pure CNO product. Electrolyses were conducted in 54 wt% $SrCO_3$, 41 wt% Li_2CO_3, and 5 wt% Na_2CO_3 in an electrolyte at 770°C and J = 0.6 A/cm^2 utilizing a stainless steel 304 carbon pot as the anode and a 168 cm^2 area brass cathode. The SEM magnifications are A: 6200x, B: 500x, C: 5,000x, D: 6,200x, E: 20,000x, and F: 110,000x magnification. CC licensee open source reference [5].

Electrode (and electrolyte additive) composition variation has been used to grow a number of other GNC allotropes from CO_2 [1-10]. These include carbon nano-bamboo, carbon nano-pearl, graphene from nanocarbon platelets, carbon nanofiber, carbon nanobelt, carbon nano-tree, and other specific carbon allotrope morphologies. SEM of a range of these GNC products is presented in Figure S6. For example, a lower temperature (725°C) is typically used in the electrolytic growth of carbon nano-onions, while higher temperature (750 to 770°C) is used in the electrolytic growth of carbon nanotubes. Binary lithium carbonate mixtures can have a lower melting point. A high sodium carbonate content in a mixed sodium/lithium carbonate electrolyte and a lower electrolysis temperature (670°C) drive the formation of a graphene scaffold nanocarbon product formation. Applied electrolysis current densities generally range

from 0.03 to 0.6 A cm^{-2}. High current density (0.6 A cm^{-2} or over) is one of the principal conditions driving the formation of fascinating helical, rather than straight, carbon nanotubes.

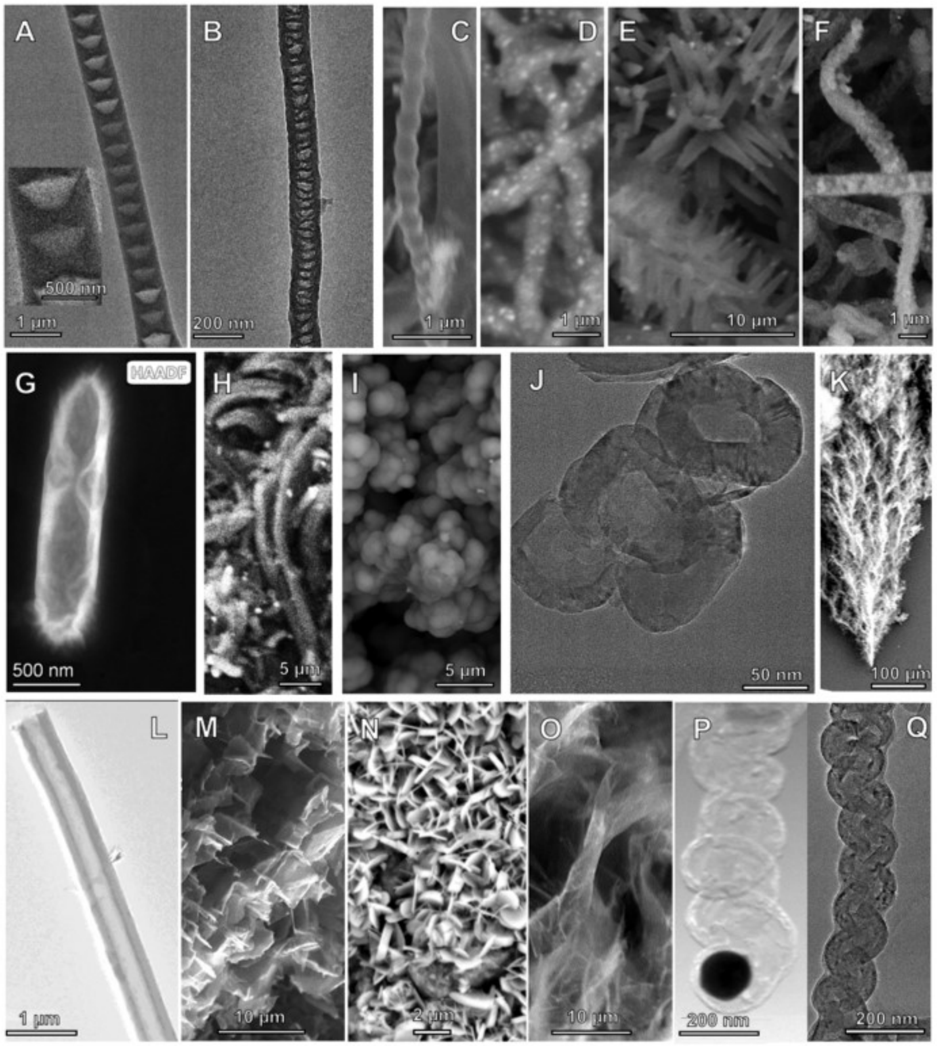

Figure S6. SEM of nanocarbon allotropes synthesized by the electrolytic splitting of CO_2 in molten carbonate. Top row (from A to F) conical CNF, nano-bamboo, nano-pearl, Ni coated CNT, nano-flower, nano-dragon. Middle row (from G to K): nano-rod, nano-belt, nano-onion, hollow nano-onion, and nano-tree. Bottom row (from L to Q) Carbon nanotube, nano-scaffold (ref. 50), nano-platelet, graphene, nano-helices. CC licensee open source reference [4].

Figure S7 presents higher resolution typical SEM, TEM and HAADF (High Angle Annular Dark-Field TEM) elemental analysis imaging of the CNT from CO2 electrolysis product, XRD and Raman characterization [3]. XRD and Raman spectra of the products are presented in Figs. S8 and S9 as previously detailed [4].

As illustrated in Figure S10, during electrolysis CO_2, either sourced directly from the air or industrial emissions, are transformed by electrolytic control to GNCs, such as a variety of forms of carbon nanotubes by electrolysis in molten carbonates. This carbon capture, utilization and sequestration (as GNCs from CO_2) is accomplished without any preconcentration of the CO_2 source. For example, the 0.04% CO_2 in the air, the 5% CO_2 from and industrial flue emission (from a 860 MW Natural gas combined cycle power plant, and from a 98% CO_2 produced from that emission using a conventional liquid amine sorbent) [1-10] all separately produce high quality graphene nanocarbons by direct feed of these various sources for this transition metal nucleated electrolytic molten carbonate CO_2 splitting process.

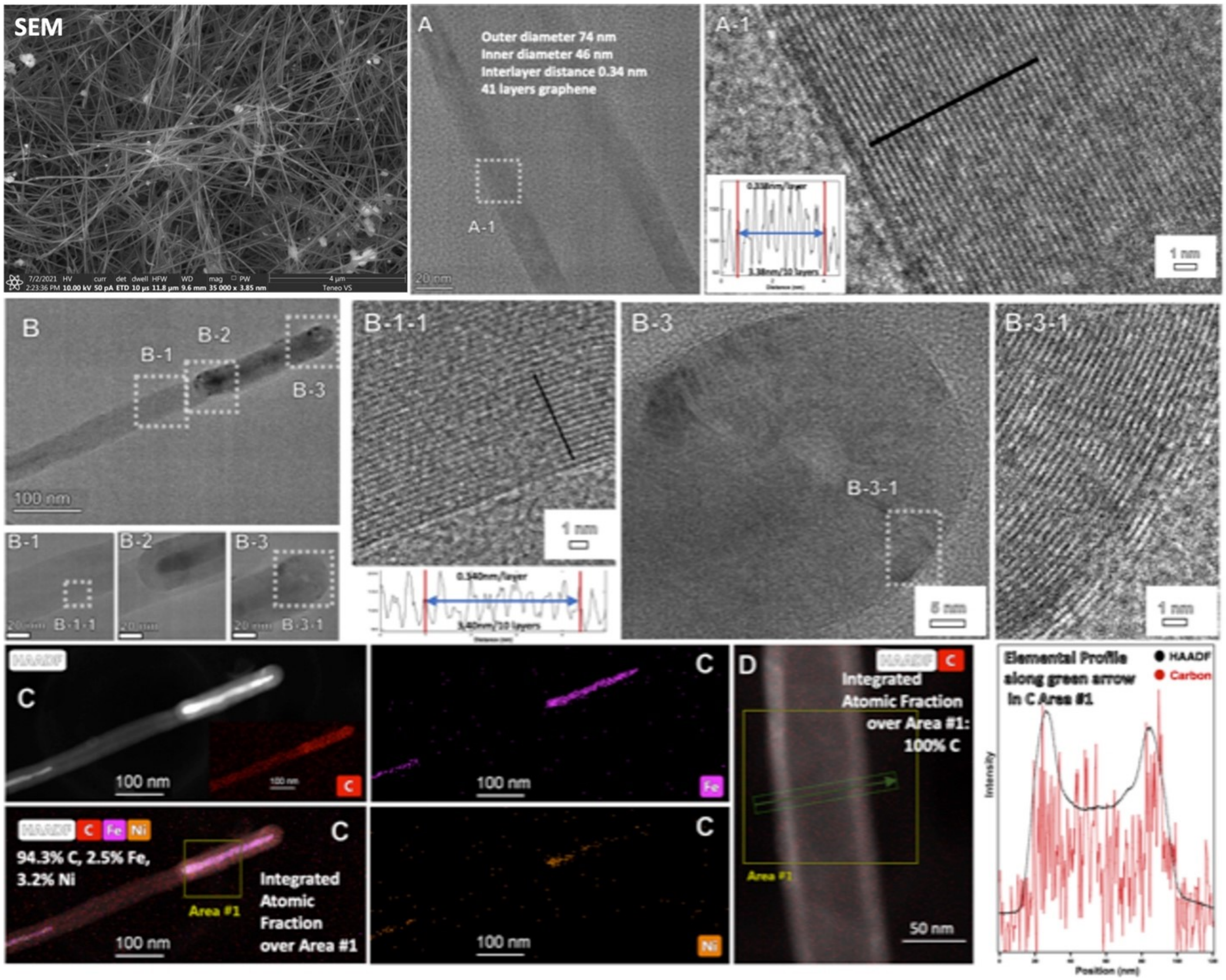

Figure S7. SEM TEM and HAADF of the synthesis product of high purity, high yield carbon nanotubes by electrolytic splitting of CO_2 in 770°C Li_2CO_3. The SEM has a scale bar of 5 µm. Panels B are TEM with scale bars decreasing from 100, 20 nm, 5 and 1 nm. Bottom rows

panels C are HAADF elemental analyses with scale bars decreasing from 100 to 50 nm, and in the bottom right a HAADF elemental carbon profile analysis of the carbon nanotube cross section. . CC licensee open source reference [3].

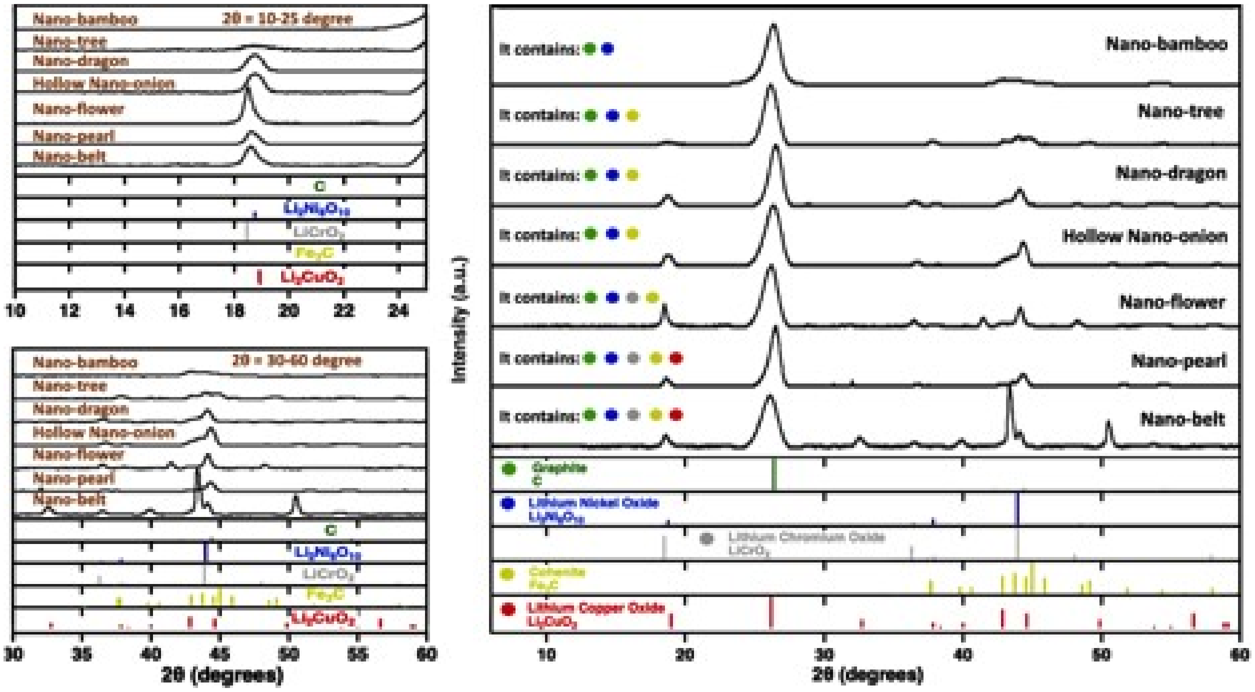

Figure S8. XRD of the synthesis product consisting of various labeled unusual nanocarbon morphologies synthesized by the electrolytic splitting of CO_2 in 770°C Li_2CO_3 with a variety of systematically varied electrochemical conditions. . CC licensee open source reference [4].

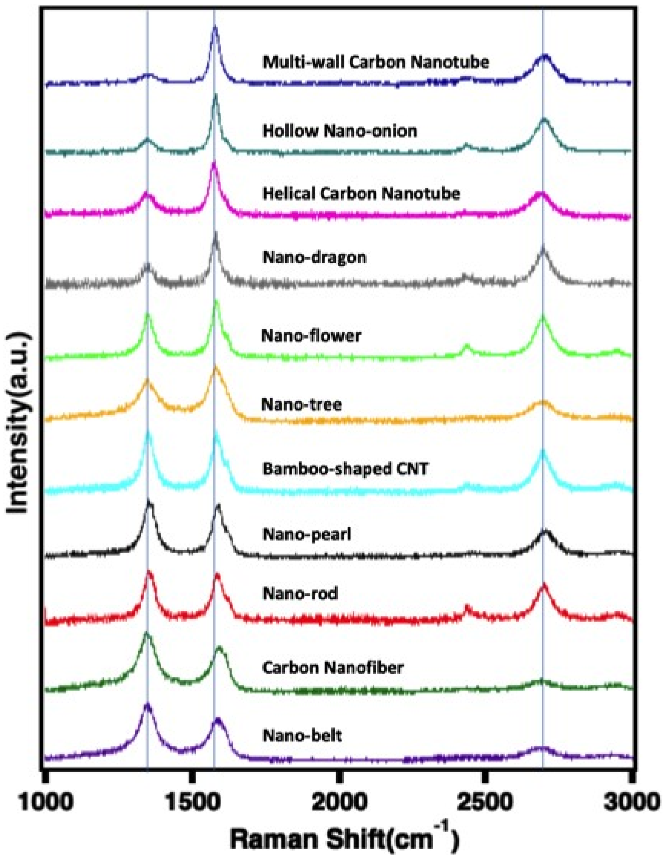

Figure S9. Raman of the synthesis product consists of various labeled GNCs and packed carbon nanotube assemblies

synthesized by the electrolytic splitting of CO_2 in 770°C Li_2CO_3 with a variety of systematically varied electrochemical conditions. . CC licensee open source reference [4].

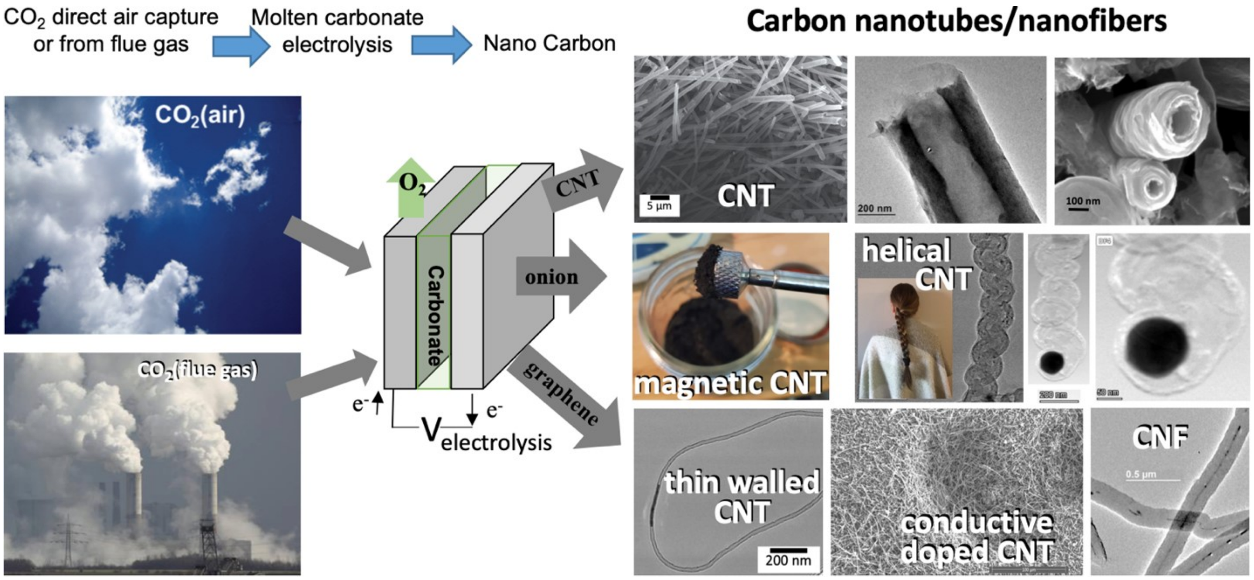

Figure S10. The CO_2 to graphene nanocarbon process. CO_2 sourced either from an anthropogenic source (CCUS) or from the air (DAC), panels is directly transformed by molten electrolysis into graphene nanocarbons, panel F. The morphology of the graphene nanocarbon is determined by tuning the electrochemical conditions of the molten carbonate CO_2 electrolysis,.

References

[1] J. Ren, F.-F. Li, J. Lau, L. Gonzalez-Urbina,S. Licht, One-pot synthesis of carbon nanofibers from CO2. Nano Lett. 15 (2015) 6142–6148. DOI: 10.1021/jp9044644.

[2] X. Liu, J. Ren, G. Licht, X. Wang and S. Licht, Carbon nano-onions made directly from CO_2 by molten electrolysis for greenhouse gas mitigation, Adv. *Sustain. Syst*., 2019, **3**, 1900056, DOI: 10.1002/adsu.201900056.

[3] X. Liu, G. Licht, S. Licht, Controlled Transition Metal Nucleated Growth of Carbon Nanotubes by Molten Electrolysis of CO_2 Catalysts 12 (2022) 137. https://doi.org/10.3390/catal12020137.

[4] X. Liu, G. Licht, X. Wang, S. Licht, Controlled Growth of Unusual Nanocarbon Allotropes by Molten Electrolysis of CO_2. Catalysts 12 (2022) 137. https://doi.org/10.3390/catal12020125.

[5] G. Licht, K. Hofstetter and S. Licht, A new electrolyte for molten carbonate decarbonization, *Nature – Communications Chemistry*, 7 (2024) 211. https://doi.org/10.1038/s42004-024-01306-z.

[6] X. Liu, G. Licht, S. Licht, The green synthesis of exceptional braided, helical carbon nanotubes and nanospiral platelets made directly from CO_2. *Mat. Today, Che.,m* 22 (2021) 100529.

[7] X. Wang, G. Licht, X. Liu and S. Licht, One pot facile transformation of CO_2 to an unusual 3-D nano-scaffold morphology of carbon, *Sci. Rep.*, 10 (2020) 21518, DOI: 10.1038/s41598-020-78258-6.

[8] X. Wang, X. Liu, G. Licht and S. Licht, Calcium metaborate induced thin walled carbon nanotube syntheses from CO_2 by molten carbonate electrolysis, *Sci. Rep*, 10 (2020) 15146, https://DOI.org/10.1038/s41598-020-71644-0.

[9] X. Wang, F. Sharif, X. Liu, G. Licht, M. Lefler and S. Licht, Magnetic carbon nanotubes: Carbide nucleated electrochemical growth of ferromagnetic CNTs. J. CO_2 Util. 40 (2020) 101218. https://DOI.org/10.1016/j.jcou.2020.101218.

[10] M. Johnson, J. Ren, M. Lefler, G. Licht, J. Vicini X. Liu, and S. Licht, Carbon nanotube wools made directly from CO_2 by molten electrolysis: Value driven pathways to carbon dioxide greenhouse gas mitigation, *Mater. Today, Energy*, 5 (2017) 230–236, DOI: 10.1016/j.mtener.2017.07.003.

[11] X. Liu, X. Wang, G. Licht and S. Licht, Transformation of the greenhouse gas carbon dioxide to graphene, *J. CO_2 Util.*, 236 (2020) 288–294, DOI: 10.1016/j.jcou.2019.11.019.
